# Aspects of telework associated with mental distress among labor court workers during the COVID-19 pandemic

**DOI:** 10.47626/1679-4435-2025-1336

**Published:** 2025-07-23

**Authors:** Francielle Barbosa Prado, Sergio Roberto de Lucca

**Affiliations:** 1 Department of Public Health, Faculty of Medical Sciences, Universidade Estadual de Campinas, Campinas, SP, Brazil

**Keywords:** occupational health, teleworking, public administration, COVID-19, Judiciary., | saúde ocupacional, teletrabalho, administração pública, COVID-19, Poder Judiciário.

## Abstract

**Introduction:**

The abrupt imposition of teleworking during the COVID-19 pandemic brought challenges that negatively affected the mental health of workers.

**Objectives:**

To identify aspects of telework and individual characteristics associated with mental distress among Brazilian labor court staff during the COVID-19 pandemic.

**Methods:**

This cross-sectional study included 1,028 workers. Independent variables were assessed using a sociodemographic and occupational questionnaire and a Likert scale instrument to measure participant perceptions about telework. Mental distress was assessed using the Self-Reporting Questionnaire. The relationship between variables was assessed with Pearson’s chi-square test or Fisher’s exact test. The association between the independent variables and the outcome was analyzed through logistic regression.

**Results:**

Mental distress was identified in 37.3% of the participants. The variables associated with the outcome were: women, not living with a partner, living with a care-dependent person, agreeing that telework has led to increased family conflict and loneliness. Mental distress was also associated with neutrality or agreement with statements on: difficulty with self-discipline, difficulty disconnecting from work, and feelings of guilt.

**Conclusions:**

The characteristics of individual workers and of telework are potential contributing factors to mental distress among teleworkers, indicating the relevance of preventive initiatives for this population.

## Abstract (pt)

**Introdução:**

A imposição abrupta do teletrabalho durante a pandemia da covid-19 trouxe desafios que implicaram prejuízos à saúde mental dos trabalhadores.

**Objetivos:**

Identificar aspectos do teletrabalho e características individuais associados ao sofrimento psíquico de servidores de um órgão da Justiça do Trabalho durante a pandemia da covid-19.

**Métodos:**

Foi realizado um estudo transversal com 1.028 servidores. As variáveis independentes foram avaliadas por meio de um questionário com questões sociodemográficas e ocupacionais, além de uma escala Likert com questões referentes a percepções sobre o teletrabalho. O sofrimento psíquico foi mensurado através do Self-Reporting Questionnaire. As relações entre as variáveis foram avaliadas pelo teste qui-quadrado de Pearson ou pelo teste exato de Fischer. Foi utilizada regressão logística para testar associações entre as variáveis independentes e o desfecho.

**Resultados:**

A prevalência de sofrimento psíquico foi de 37,3%. As variáveis associadas ao desfecho foram: sexo feminino, não ter companheiro(a), viver com dependentes de cuidados, concordar que o teletrabalho trouxe aumento dos conflitos familiares e sensação de solidão. Além disso, associaram-se ao sofrimento psíquico as respostas “neutro” ou “concordo” para: dificuldade para se disciplinar, dificuldade para se desconectar dos assuntos de trabalho e sentimento de culpa.

**Conclusões:**

Características individuais e do teletrabalho são potenciais fatores contribuintes para a ocorrência de sofrimento psíquico entre os teletrabalhadores, confirmando a relevância de ações preventivas direcionadas a este público.

## INTRODUCTION

Telework, defined as all work carried out remotely using information and communication technologies, is becoming increasingly common worldwide.^1^ In Brazil, although remote work was first implemented in private businesses, public service followed suit.^2^

The COVID-19 pandemic has had a major impact on this trend, accelerating its growth.^3,4^ In 2022, 36% of the world’s population was teleworking, albeit partially, in contrast to 16% before the pandemic.^1^ Although telework provided greater protection against the virus, it also affected workers’ personal lives.^5^

Despite its positive aspects, such as flexible working hours, no commuting, and greater balance between personal and professional lives, telework can negatively affect mental health. Factors such as social and professional isolation, increased working hours, and conflict between professional and personal life^5-9^ can cause stress, anxiety, depression, and other mental disorders.^10,11^

During the pandemic, the lack of adequate infrastructure and overlapping domestic and professional tasks made it difficult for many workers to adapt.^5,12-14^ A study of teleworkers during the pandemic found an association between long working hours and stress, while low control over working time was related to depression, anxiety, and stress.^10^ A survey of labor court workers in the same period found a mental distress prevalence of 45.38% among judges and 36.94% among civil servants. These rates were associated with highly demanding work, during a period of lower social support.^11^

As COVID-19 infection rates dropped, many companies returned to in-person work or a hybrid model, which changed the psychosocial factors of telework, with greater social support on the one hand, but less perceived autonomy on the other.^11^

The conversion to telework during the pandemic involved distinct specificities from those under normal conditions, which can affect worker health.^10^

## OBJECTIVES

Considering the continuing post-pandemic trend toward telework and the importance of understanding its psychosocial impacts, the objective of this study was to identify aspects of telework and individual characteristics associated with mental distress among labor court employees during the COVID-19 pandemic.

## METHODS

This cross-sectional study was conducted in the second largest regional labor court in Brazil, which employs 3,481 staff. Court employees who were working remotely, either completely or partially, were invited to participate in the survey via institutional email that included a link to the questionnaires. The 68 security employees were excluded due to the specific nature of their activities.

Employees who indicated that they did not telework completely or partially were excluded, as were individuals who failed to respond to at least 20 items in the questionnaires.

Thus, given these eligibility criteria, sample size calculation was based on a population of 3,413 individuals. Assuming a sampling error of 5% and a significance level of 5%, the sample size was calculated at 346, but considering a non-response rate of 20%, at least 416 employees would be required. However, the final convenience sample included 1,028 participants, or 30.12% of the total population.

At the time the questionnaires were administered, vaccination against COVID-19 had not yet begun and, in the state of São Paulo, organizations were operating according to the São Paulo Plan, which involves fairly stringent measures to restrict movement depending on the public health situation in each municipality.^15^ Thus, during this period, the investigated court units were operating in accordance with this plan.^16^

Data were collected in February and March 2021 through self-administered instruments available online at SurveyMonkey, an online questionnaire and survey platform (https://www.surveymonkey.com/). All employees were invited to participate in the survey via institutional email.

A demographic and occupational questionnaire was designed specifically for this study. Since we could find no validated instruments to assess the characteristics or positive and negative aspects of telework, we also developed a 5-point Likert scale instrument based on common telework variables found in the literature. Before making the questionnaire available, it was pilot tested for validation. Eight civil servants from the investigated agency participated in the test, in addition to five psychology professionals from other judiciary agencies, who suggested changes that were analyzed and incorporated. However, no reliability analyses of the scale were performed.

In the end, 3 strata were considered for analysis: agreement (options 4 and 5), neutrality (option 3) and disagreement (options 1 and 2). These strata were considered as “best condition,” “worst condition” or “neutral condition,” considering the favorable or unfavorable influence of each variable.

The outcome was measured using the Self-Reporting Questionnaire, which detects common mental disorders indicative of mental distress. Developed by Harding et al.^17^ and validated in Brazil by Mari & Willians,^18^ the instrument consists of 20 questions covering physical and psychological symptoms, with a dichotomous (yes/no) response scale. The instrument is intended to detect symptoms suggestive of mental distress without making diagnostic discriminations. Each affirmative response is scored as 1 point, and the cut-off point for suspected mental distress was considered ≥ 7 points.^18^

The data were analyzed in Stata 18. For descriptive analyses, simple and relative frequencies were used, in addition to measures of central tendency and dispersion for categorical and continuous variables, respectively. The prevalence of mental distress was calculated, and bivariate analysis between groups with and without the outcome was performed. For categorical variables, Pearson’s chi-square test or Fisher’s exact test were applied, depending on the distribution of the variables, with the significance level set at 5%. Through logistic regression analysis, the unadjusted odds ratio and 95%CI were obtained. The measure of association was converted to prevalence ratio (PR) using the Zhang & Yu technique.^19^

Hierarchical analysis of the factors associated with mental distress was performed after the independent variables were selected according to epidemiological importance and a theoretical-conceptual model had been constructed. A significance level ≤ 20% in bivariate analysis was also considered. Collinearity between independent variables was assessed using the Pearson correlation coefficient with covariance matrix. In the first block (distal hierarchical level), socioeconomic variables were tested. In the second block (intermediate hierarchical level), variables related to the work routine were tested. In the third block (proximal hierarchical level), demographic variables, work routines, and stressors were tested.

Adjustment variables for the multiple regression analysis were selected based on a theoretical-conceptual model of causality between the independent variables and mental distress, in addition to the hierarchical level analysis. Logistic regression analysis identified factors associated with mental distress, obtaining the adjusted odds ratio and 95%CI. The measure of association was converted to adjusted PR using the Zhang & Yu technique.^19^ After the modeling process, the quality of each analysis block was assessed using the Hosmer & Lemeshow test^20^ with a 10% significance level.

The research was authorized by justice department management and was approved by the Universidade Estadual de Campinas Research Ethics Committee (opinion 4,415,662/2020). Participants could only join the study after providing written informed consent on the study’s home page.

## RESULTS

Of the 3,413 employees who were emailed about participation, 1,173 opened the link and signed the consent form, of whom 24 subsequently declined to participate. Of the 1,149 employees who agreed to participate, 9 reported that they were not fully or partially teleworking at the time of the survey and were excluded. Of the remaining 1,140 participants, 12 failed to respond to at least 20 survey questions and were also excluded. Thus, data from 1,028 participants were analyzed.

The prevalence of mental distress was 37.26%. The mean participant age was 46.18 years (standard deviation [SD] = 8.40). Women were 1.29 times (95%CI 1.09-1.54) more likely to experience mental distress than men. The prevalence of mental distress was 1.21 times higher (95%CI 1.02-1.44) among participants who did not live with a partner than those who did, and it was 1.28 times higher (95%CI 1.09-1.50) among those who lived with a care-dependent person than those who did not. A lack of training prior to starting telework and a greater perceived workload increased the probability of mental distress by 28% (95%CI 1.03-1.61) and 70% (95%CI 1.42-2.04), respectively (Table 1).

**Table 1:** Prevalence of mental distress according to sociodemographic and occupational characteristics of the study population, state of São Paulo, 2021

Variable	Mental distress		p-value	PR	95%CI
	No n (%)	Yes n (%)			
Age (years)					
20-39	161 (60.98)	103 (39.02)	-	1.00	-
40-49	225 (62.67)	134 (37.33)	0.66	0.95	0.78-1.17
≥ 50	255 (64.23)	142 (35.77)	0.39	0.91	0.75-1.12
Mean	46.42 (8.64)	45.79 (7.99)	-	-	-
Sex					
Male	273 (68.42)	126 (31.58)	-	1.00	
Female	368 (58.97)	256 (41.03)	0.02	1.29	1.09-1.54
Marital status					
With partner	484 (64.71)	264 (35.29)	-	1.00	
Without partner	156 (57.14)	117 (42.86)	0.02	1.21	1.02-1.44
Lives with people who depend on routine care					
No	410 (66.56)	206 (33.44)	-	1.00	
Yes	234 (57.07)	176 (42.93)	0.00	1.28	1.09-1.50
Holds a management position					
No	489 (62.29)	296 (37.71)	-	1.00	
Yes	155 (64.58)	85 (35.42)	0.52	0.93	0.77-1.13
Received training prior to beginning telework					
Yes	145 (69.71)	63 (30.29)	-	1.00	
No	499 (61.00)	319 (39.00)	0.02	1.28	1.03-1.61
In teleworking. consider that the workload					
No change	310 (73.29)	113 (26.71)	-	1.00	
Increased	306 (54.45)	256 (45.55)	0.00	1.70	1.42-2.04
Decreased	28 (70.00)	12 (30.00)	0.65	1.12	0.68-1.85

PR = prevalence ratio; 95%CI = 95% confidence interval.

Regarding the item “The nature of the tasks is adaptable to telework,” those who disagreed and those who were neutral were 1.67 (95%CI 1.28-2.18) and 2.23 (95%CI 1.62-3.07) times more likely to experience mental distress, respectively, than those who agreed. People who did not wish to continue working remotely after the pandemic were also 89% (95%CI 1.60-2.23) more likely to experience mental distress than those did (Table 2).

**Table 2:** Prevalence of mental distress according to perceived aspects of telework, state of São Paulo, 2021

Characteristics of telework	Mental distress		P-value	PR	95%CI
	No n (%)	Yes n (%)			
Task completion depends on information from other people					
Disagree	174 (68.77)	79 (31.23)	-	1.00	
Agree	420 (60.17)	278 (39.83)	0.016	1.27	1.04-1.56
Neutral	50 (67.57)	24 (32.43)	0.843	1.04	0.71-1.51
The nature of the tasks is adaptable to telework					
Agree	626 (64.14)	350 (35.86)	-	1.00	
Disagree	16 (40.00)	24 (60.00)	0.003	1.67	1.28-2.18
Neutral	2 (20.00)	8 (80.00)	0.013	2.23	1.62-3.07
If it were up to you, you would continue working remotely after the pandemic.					
Agree	566 (68.44)	261 (31.56)	-	1.00	
Disagree	58 (40.28)	86 (59.72)	0.000	1.89	1.60-2.23
Neutral	20 (36.36)	35 (63.64)	0.000	2.02	1.61-2.52

PR = prevalence ratio; 95%CI = 95% confidence interval.

Regarding the perceived consequences of telework, 4 of the 15 advantages were not significantly associated (p < 0.05) with mental distress: saving time previously spent commuting, increased concentration, greater work autonomy, and fewer unwanted personal interactions with coworkers. However, disagreeing with statements about the other advantages was significantly associated (p < 0.05) with mental distress. The probability of mental distress was 2.82 times higher (95%CI 2.36-3.36) among those who disagreed that telework reduces stress, 2.28 times higher (95%CI 1.93-2.69) among those who disagreed that telework increases job satisfaction, and 2.15 times higher (95%CI 1.82-2.54) among those who disagreed that telework increases time spent with family and increases productivity (Table 3).

**Table 3:** Prevalence of mental distress according to the perceived advantages of telework, state of São Paulo, 2021

Advantages of telework	Mental distress		P-value	PR	95%CI
	No n (%)	Yes n (%)			
Can dress more comfortably					
Agree	612 (63.62)	350 (36.38)	-	1.00	
Disagree	12 (40.00)	18 (60.00)	0.01	1.64	1.21-2.23
Neutral	14 (58.33)	10 (41.67)	0.59	1.14	0.70-1.85
Save time previously spent commuting					
Agree	588 (64.97)	317 (35.03)	-	1.00	
Disagree	15 (32.61)	31 (67.39)	0.00	1.92	1.54-2.39
Neutral	13 (54.17)	11 (45.83)	0.27	1.31	0.83-2.04
Lower costs					
Agree	579 (66.17)	296 (33.83)	-	1.00	
Disagree	46 (41.07)	66 (58.93)	0.00	1.74	1.45-2.08
Neutral	10 (45.45)	12 (54.55)	0.04	1.61	1.01-2.38
Less exposure to violence					
Agree	533 (65.24)	284 (34.76)	-	1.00	
Disagree	36 (50.70)	35 (49.30)	0.01	1.41	1.09-1.83
Neutral	40 (49.38)	41 (50.62)	0.00	1.45	1.15-1.84
Flexible work hours and location					
Agree	591 (66.18)	302 (33.82)	-	1.00	
Disagree	36 (34.29)	69 (65.71)	0.00	1.94	1.64-2.29
Neutral	9 (52.94)	8 (47.06)	0.26	1.39	0.83-2.32
More time spent with family					
Agree	565 (66.86)	280 (33.14)	-	1.00	
Disagree	24 (28.57)	60 (71.43)	0.00	2.15	1.82-2.54
Neutral	32 (50.00)	32 (50.00)	0.00	1.50	1.16-1.96
Easier to reconcile personal and professional commitments					
Agree	576 (66.51)	290 (33.49)	-	1.00	
Disagree	34 (33.33)	68 (66.67)	0.00	1.99	1.68-2.35
Neutral	21 (46.67)	24 (53.33)	0.00	1.59	1.19-2.12
Personalized work environment					
Agree	554 (67.15)	271 (32.85)	-	1.00	
Disagree	44 (40.37)	65 (59.63)	0.00	1.81	1.51-2.10
Neutral	30 (43.48)	39 (56.52)	0.00	1.72	1.36-2.16
Increased productivity					
Agree	545 (69.07)	244 (30.93)	-	1.00	
Disagree	44 (33.33)	88 (66.67)	0.00	2.15	1.83-2.52
Neutral	43 (47.48)	47 (52.22)	0.00	1.68	1.35-2.11
Easier to concentrate					
Agree	519 (70.61)	216 (29.39)	-	1.00	
Disagree	83 (37.05)	141 (62.95)	0.00	2.14	1.84-2.49
Neutral	36 (61.02)	23 (38.98)	0.12	1.32	0.95-1.86
Greater work autonomy					
Agree	484 (67.79)	230 (32.21)	-	1.00	
Disagree	77 (42.31)	105 (57.69)	0.00	1.79	1.52-2.11
Neutral	54 (62.07)	33 (37.93)	0.28	1.17	0.88-1.57
Fewer unwanted personal interactions with coworkers					
Agree	320 (62.75)	190 (37.25)	-	1.00	
Disagree	109 (57.67)	80 (42.33)	0.22	1.13	0.92-1.38
Neutral	88 (62.86)	52 (37.14)	0.98	0.99	0.78-1.27
Greater job satisfaction					
Agree	431 (71.95)	168 (28.05)	-	1.00	
Disagree	73 (35.96)	130 (64.04)	0.00	2.28	1.93-2.69
Neutral	116 (61.05)	74 (38.95)	0.00	1.38	1.11-1.72
More leisure time					
Agree	410 (73.35)	149 (26.65)	-	1.00	
Disagree	144 (43.77)	185 (56.23)	0.00	2.10	1.78-2.49
Neutral	71 (64.55)	39 (35.45)	0.06	1.33	0.99-1.77
Less stress					
Agree	432 (77.84)	123 (22.16)	-	1.00	
Disagree	136 (37.47)	227 (62.53)	0.00	2.82	2.36-3.36
Neutral	63 (67.02)	31 (32.98)	0.02	1.48	1.07-2.06

PR = prevalence ratio; 95%CI = 95% confidence interval.

Agreeing with statements about the disadvantages of telework was associated with mental distress. The probability of mental distress was 4.86 (95%CI 3.70-6.38) times higher among those who agreed that telework leads to difficulty disconnecting from work, 4.51 (95%CI 3.29-6.19) times higher among those who agreed that telework leads to increased musculoskeletal pain, and 4.12 (95%CI 3.25-5.23) times higher among those who agreed that telework leads to intrusion of work into personal life and personal life into work (Table 4).

**Table 4:** Prevalence of mental distress according to the perceived disadvantages of telework, state of São Paulo, 2021

Disadvantages of telework	Mental distress		P-value	PR	95%CI
	No n (%)	Yes n (%)			
Uncompensated home office expenses					
Disagree	126 (84.00)	24 (16.00)	-	1.00	
Agree	460 (57.36)	342 (42.64)	0.00	2.66	1.83-3.88
Neutral	46 (75.41)	15 (24.59)	0.14	1.53	0.86-2.72
Emotional distancing from coworkers					
Disagree	130 (83.33)	26 (16.67)	-	1.00	
Agree	455 (58.56)	322 (41.44)	0.00	2.48	1.73-3.56
Neutral	48 (62.34)	29 (37.66)	0.00	2.26	1.43-3.55
Unforeseen events due to technical difficulties					
Disagree	206 (83.74)	40 (16.26)	-	1.00	
Agree	372 (53.91)	318 (46.09)	0.00	2.83	2.11-3.80
Neutral	51 (69.86)	22 (30.14)	0.01	1.85	1.18-2.90
Increased working hours					
Disagree	229 (82.97)	47 (17.03)	-	1.00	
Agree	327 (52.66)	294 (47.34)	0.00	2.78	2.11-3.65
Neutral	68 (67.33)	33 (32.67)	0.00	1.91	1.30-2.81
Increased musculoskeletal pain					
Disagree	270 (88.24)	36 (11.76)	-	1.00	
Agree	281 (46.83)	319 (53.19)	0.00	4.51	3.29-6.19
Neutral	72 (75.79)	23 (24.21)	0.00	2.05	1.28-3.29
Difficulty disconnecting from work					
Disagree	360 (88.02)	49 (11.98)	-	1.00	
Agree	219 (41.71)	306 (58.29)	0.00	4.86	3.70-6.38
Neutral	45 (62.50)	27 (37.50)	0.00	3.13	2.10-4.65
Excessive use of virtual communication					
Disagree	328 (79.04)	87 (20.96)	-	1.00	
Agree	219 (47.51)	242 (52.49)	0.00	2.50	2.03-3.07
Neutral	69 (60.53)	45 (39.47)	0.00	1.88	1.40-2.52
Intrusion of work into personal life and personal life into work					
Disagree	374 (85.39)	64 (14.61)	-	1.00	
Agree	190 (39.67)	289 (60.33)	0.00	4.12	3.25-5.23
Neutral	52 (71.23)	21 (28.77)	0.00	1.96	1.28-3.01
Feelings of loneliness					
Disagree	376 (83.74)	73 (16.26)	-	1.00	
Agree	198 (41.60)	278 (58.40)	0.00	3.59	2.87-4.49
Neutral	52 (66.67)	26 (33.33)	0.00	2.05	1.40-2.99
Feelings of guilt: not being able to pay attention to nearby family members or resting instead of working					
Disagree	412 (83.74)	80 (16.26)	-	1.00	
Agree	134 (35.83)	240 (64.17)	0.00	3.94	3.18-4.89
Neutral	41 (57.75)	30 (42.25)	0.00	2.59	1.85-3.64
Difficulty reconciling domestic and professional activities					
Disagree	412 (80.78)	98 (19.22)	-	1.00	
Agree	156 (38.52)	249 (61.48)	0.00	3.19	2.63-3.88
Neutral	53 (63.86)	30 (36.14)	0.00	1.88	1.34-2.63
Lack of adequate work space					
Disagree	414 (75.27)	136 (24.73)	-	1.00	
Agree	176 (45.24)	213 (54.76)	0.00	2.21	1.86-2.62
Neutral	32 (60.38)	21 (39.62)	0.02	1.60	1.11-2.30
Loss of visibility and career opportunities					
Disagree	347 (76.26)	108 (23.74)	-	1.00	
Agree	125 (49.21)	129 (50.79)	0.00	2.13	1.74-2.62
Neutral	109 (53.69)	94 (46.31)	0.00	1.95	1.56-2.43
Difficulty with self-discipline and maintaining a work routine					
Disagree	479 (75.91)	152 (24.09)	-	1.00	
Agree	118 (38.82)	186 (61.18)	0.00	2.53	2.15-2.99
Neutral	31 (44.93)	38 (55.07)	0.00	2.28	1.77-2.94
Increased family conflict					
Disagree	485 (76.26)	151 (23.74)	-	1.00	
Agree	65 (31.40)	142 (68.60)	0.00	2.88	2.44-3.41
Neutral	44 (49.44)	45 (50.56)	0.00	2.13	1.66-2.72

PR = prevalence ratio; 95%CI = 95% confidence interval.

In the hierarchical multivariate analysis (Table 5), among the distal factors (first block), there was an association between mental distress and female sex, living without a partner, and living with a care-dependent person. These variables increased the probability of mental distress by 23% (95%CI 1.04-1.47), 25% (95%CI 1.05-1.49), and 30% (95%CI 1.10-1.54), respectively.

**Table 5:** Logistic regression model showing the association between telework variables and mental distress, state of São Paulo, 2021

Disadvantages of teleworking	Mental distress		P-value	PR	95%CI
	No n (%)	Yes n (%)			
Uncompensated home office expenses					
Disagree	126 (84.00)	24 (16.00)	-	1.00	
Agree	460 (57.36)	342 (42.64)	0.00	2.66	1.83-3.88
Neutral	46 (75.41)	15 (24.59)	0.14	1.53	0.86-2.72
Emotional distancing from coworkers					
Disagree	130 (83.33)	26 (16.67)	-	1.00	
Agree	455 (58.56)	322 (41.44)	0.00	2.48	1.73-3.56
Neutral	48 (62.34)	29 (37.66)	0.00	2.26	1.43-3.55
Unforeseen events due to technical difficulties					
Disagree	206 (83.74)	40 (16.26)	-	1.00	
Agree	372 (53.91)	318 (46.09)	0.00	2.83	2.11-3.80
Neutral	51 (69.86)	22 (30.14)	0.01	1.85	1.18-2.90
Increased working hours					
Disagree	229 (82.97)	47 (17.03)	-	1.00	
Agree	327 (52.66)	294 (47.34)	0.00	2.78	2.11-3.65
Neutral	68 (67.33)	33 (32.67)	0.00	1.91	1.30-2.81
Increased musculoskeletal pain					
Disagree	270 (88.24)	36 (11.76)	-	1.00	
Agree	281 (46.83)	319 (53.19)	0.00	4.51	3.29-6.19
Neutral	72 (75.79)	23 (24.21)	0.00	2.05	1.28-3.29
Difficulty disconnecting from work					
Disagree	360 (88.02)	49 (11.98)	-	1.00	
Agree	219 (41.71)	306 (58.29)	0.00	4.86	3.70-6.38
Neutral	45 (62.50)	27 (37.50)	0.00	3.13	2.10-4.65
Excessive use of virtual communication					
Disagree	328 (79.04)	87 (20.96)	-	1.00	
Agree	219 (47.51)	242 (52.49)	0.00	2.50	2.03-3.07
Neutral	69 (60.53)	45 (39.47)	0.00	1.88	1.40-2.52
Intrusion of work into personal life and personal life into work					
Disagree	374 (85.39)	64 (14.61)	-	1.00	
Agree	190 (39.67)	289 (60.33)	0.00	4.12	3.25-5.23
Neutral	52 (71.23)	21 (28.77)	0.00	1.96	1.28-3.01
Feelings of loneliness					
Disagree	376 (83.74)	73 (16.26)	-	1.00	
Agree	198 (41.60)	278 (58.40)	0.00	3.59	2.87-4.49
Neutral	52 (66.67)	26 (33.33)	0.00	2.05	1.40-2.99
Feelings of guilt: not being able to pay attention to nearby family members or resting instead of working					
Disagree	412 (83.74)	80 (16.26)	-	1.00	
Agree	134 (35.83)	240 (64.17)	0.00	3.94	3.18-4.89
Neutral	41 (57.75)	30 (42.25)	0.00	2.59	01.85-3.64
Difficulty reconciling domestic and professional activities					
Disagree	412 (80.78)	98 (19.22)	-	1.00	
Agree	156 (38.52)	249 (61.48)	0.00	3.19	2.63-3.88
Neutral	53 (63.86)	30 (36.14)	0.00	1.88	1.34-2.63
Lack of adequate work space					
Disagree	414 (75.27)	136 (24.73)	-	1.00	
Agree	176 (45.24)	213 (54.76)	0.00	2.21	1.86-2.62
Neutral	32 (60.38)	21 (39.62)	0.02	1.60	1.11-2.30
Loss of visibility and career opportunities					
Disagree	347 (76.26)	108 (23.74)	-	1.00	
Agree	125 (49.21)	129 (50.79)	0.00	2.13	1.74-2.62
Neutral	109 (53.69)	94 (46.31)	0.00	1.95	1.56-2.43
Difficulty with self-discipline and maintaining a work routine					
Disagree	479 (75.91)	152 (24.09)	-	1.00	
Agree	118 (38.82)	186 (61.18)	0.00	2.53	2.15-2.99
Neutral	31 (44.93)	38 (55.07)	0.00	2.28	01.77-2.94
Increased family conflict					
Disagree	485 (76.26)	151 (23.74)	-	1.00	
Agree	65 (31.40)	142 (68.60)	0.00	2.88	2.44-3.41
Neutral	44 (49.44)	45 (50.56)	0.00	2.13	1.66-2.72

PR = prevalence ratio; 95%CI = 95% confidence interval.

After adjusting for variables in the second block and in the first block, the following remained associated with the mental distress: neutrality or agreement about difficulty with self-discipline and maintaining a work routine and difficulty disconnecting from work. Agreeing with the latter statement increased the probability of mental distress by 3 times (95%CI 2.29-4.10), while agreeing that telework leads to feelings of loneliness increased the probability of mental distress by 2 times (95%CI 1.58-2.54).

The following variables of the third (proximal) block remained associated with mental distress after adjusting for the two more distal blocks: neutrality (PR = 1.66; 95%CI 1.17-2.36) or agreement (PR = 1.86; 95%CI 1.43-2.41) about feelings of guilt; neutrality (PR = 1.38; 95%CI 1.06-1.79) or agreement (PR = 1.28; 95%CI 1.07-1.53) about difficulty with self-discipline and maintaining a work routine; neutrality (PR = 2.08; 95%CI 1.36-3.21) or agreement (PR = 2.60; 95%CI 1.84-3.68) about difficulty disconnecting from work; and agreement that teleworking leads to increased family conflict (PR = 1.21; 95%CI 1.02-1.44) and loneliness (PR = 1.62; 95%CI 1.26-2.08) (Table 5).

## DISCUSSION

Mental distress was significantly higher among women, corroborating research results that women are more susceptible to common mental disorders.^21,22^ However, it should also be considered that women suffered greater mental health impairment during the pandemic due to greater overload from asymmetrical distribution of domestic activities.^13,23,24^ As in the present study, Şentürk et al.^23^ investigated teleworkers during social distancing, finding a higher prevalence of depression, anxiety, and stress among women.

The same occurred in relation to marital status. In line with our results, studies have found a higher prevalence of common mental disorders among the divorced, separated, and widowed.^21,22^ It is also important to consider their increased isolation while social distancing measures were in effect. According to the literature, one of the main disadvantages of teleworking is loneliness,^5,8,9^ which can affect both mental health and work relationships. A study found that teleworkers who felt more isolated had less emotional commitment to the organization.^9^ Furthermore, loneliness can increase vulnerability to depression and anxiety,^21^ which was corroborated in the present study, since those who agreed that teleworking intensified feelings of loneliness were more likely to experience mental distress.

A higher prevalence of mental distress was also observed among workers who lived with a care-dependent person. This result also confirms other research results during the pandemic,^14,25^ such as Xiao et al.,^14^ who found that teleworkers with children at home had lower levels of physical and mental well-being than those who did not. However, these results are also in line with pre-pandemic studies, which found that parenthood appears to be a motivator for telework since it facilitates child care dynamics.^26,27^ Kossek et al.^26^ found that teleworkers with children had lower rates of depression than those who did not engage in telework. An important element in this context is the teleworker’s support network, with one study indicating that teleworkers with children but limited access to support have higher rates of exhaustion.^13^

In this regard, it is important to consider the exceptional conditions during the pandemic, especially reduced childcare support networks, such as schools, nannies, and family members.^14,24^ Workers began to reconcile these obligations through remote work, although Vasquez et al.^24^ found that 28.20% of men and 32.00% of women considered reconciling telework and family relationships to be difficult or very difficult. The conflict between these roles can produce negative feelings that are difficult to overcome.^13,28^ In the present study, participants who agreed that teleworking increased their feelings of guilt about not being able to pay attention to nearby family members or about resting when they could be working were more likely to experience mental distress.

The relationship between personal and professional life is a controversial topic in the literature. On the one hand, teleworking is considered to help balance family and professional life.^2,6^ On the other, it can erode the borders between these domains and generate conflict.^26,27^ Difficulty managing them appears to be related to worsening mental health,^26^ which was corroborated in our study our study because agreement that teleworking increased family conflict was associated with mental distress.

These overlapping domains can lead to difficulty disconnecting from work.^6,28^ In our results, neutrality regarding this statement was associated with mental distress, although not as strongly as agreement.

Work overload issues are closely associated with the home-work interface^28,29^ and are related to institutional demands. In addition to the difficulties brought about by the intersection of these domains, the institution’s lack of control over traditional indicators, such as attendance and compliance with schedules, could result in greater pressure for productivity.^4^

Teleworking can also complicate work schedule organization.^5,29^ People with low self-discipline may feel more anxious without the common rules of face-to-face work.^6,29^ In the present study, those who were neutral or agreed regarding “difficulty with self-discipline and maintaining a work routine” were more likely to experience mental distress. However, curiously, a neutral response was more associated with mental distress than agreement. One explanation for this could be related to worker preferences, for example, some respondents may have omitted information that could harm the continuity of telework, such as self-discipline difficulties.

The results of this study cannot be generalized, as they reflect teleworking conditions at an atypical time and include a population with specific characteristics. Furthermore, being a cross-sectional study, cause and effect relationships could not be established. Longitudinal studies on the subject, as well as studies conducted under normal circumstances, are needed.

The strengths of this study include the study population and sample size, as well as its contribution to the literature on mental health in telework. Few studies have been published on the health of labor court employees, and few studies have established associations between aspects of remote work and mental health indicators. The results of this study can help map factors that influence the mental health of teleworkers, in addition to supporting protocols for healthier telework practices.

## CONCLUSIONS

This study aimed to identify aspects of telework associated with mental distress among employees of a labor court during the COVID-19 pandemic. The findings indicated that female sex, not living with a partner, and living with a care-dependent person were associated with mental distress. The telework characteristics associated with mental distress were increased family conflict, feelings of loneliness, difficulty with self-discipline, difficulty disconnecting from work, and feelings of guilt.

This study highlights the importance of providing guidance to teleworkers about organizing the work routine, the need for scheduled rest periods, and a consistent support network. However, such guidance should be part of systematic organizational training initiatives for teleworkers and managers. What is needed are efficient communication channels that consider the synchronous or asynchronous nature of activities and, above all, the characteristics of each worker, such as sex, family dynamics, and health status.
